# Complete genome sequences of eight *Auritidibacter ignavus* strains isolated from ear infections in Germany

**DOI:** 10.1128/MRA.00666-23

**Published:** 2023-10-17

**Authors:** Tobias Bähr, Ariane Baumhögger, Gabriele Geis, Sören Gatermann

**Affiliations:** 1 Institute of Medical Laboratory Diagnostics, Bochum, Germany; 2 Department of Medical Microbiology, Ruhr University Bochum, Bochum, Germany; Rochester Institute of Technology, Rochester, New York, USA

**Keywords:** ear infections, pathogens

## Abstract

Here, we present the complete genome sequences of eight *Auritidibacter ignavus* strains isolated from clinical samples of patients with ear infections in Bochum, Germany. The sequence information will give assistance to greater knowledge about the virulence potential of this unfamiliar putative pathogen.

## ANNOUNCEMENT

*Auritidibacter ignavus* was initially isolated from a patient with fulminant otitis externa and was described by Yassin et al. in 2011 (1). Since then, the species has received little attention. Consequently, limited information on this putative agent of ear infections is available so far.

We initially detected the species in a culture negative mastoid biopsy of a patient with mastoiditis in 2021 by 16S-rRNA-PCR assay with the primer set 27f (5′-AGAGTTTGATCMTGGCTCAG-3′) and 1492r (5′-CGGTTACCTTGTTACGACTT-3′) as described by Jiang et al. (2). The nucleotide Basic Local Alignment Search Tool (BLAST) database was used for species identification. Follow-up samples grew unfamiliar colonies that were also identified via PCR. Since the species was included in Bruker’s matrix-assisted laser desorption ionization (MALDI) database in April 2022, it has been occasionally detected in our diagnostic institute.

The strains originated from clinical samples of patients with ear infections, submitted by a local hospital for diagnostic purposes. The isolates were cultivated on blood agar (bioMeriéux) and incubated for 48 h at 37°C under capnophilic conditions.

Colonies were identified by MALDI-time of flight mass spectrometry (MALDI-TOF MS) with a MALDI Biotyper instrument (Bruker).

Genomic DNA was extracted from fresh colonies using the QIAamp DNA Mini Kit (Qiagen), following the manufacturer’s protocol for Gram-positive bacteria.

Libraries for short-read sequencing were prepared using the Nextera XT DNA Library Preparation Kit (Illumina). The sequencing was performed on a MiSeqDx platform using the MiSeqDx Reagent Kit v.3 (Illumina) in a 300-bp paired-end workflow. Adapter trimming was performed by the local run manager software (Illumina). The reads were trimmed with BBDuk v.38.84 (minimum quality, Q30; minimum length, 30 bp).

For long-read sequencing, the same DNA extractions were used. Libraries were prepared with the Rapid Barcoding Kit (SQK-RBK004, Oxford Nanopore Technologies) and were sequenced on a GridION system with an R9.4.1 flow cell (Oxford Nanopore Technologies). Base calling and barcode trimming were performed using Guppy v.6.4.6. Error correction was not performed. The sequencing averagely produced 97.82-Mb data with *Q* scores of ≥10. The average *Q* score was 21.3.

The reads were assembled using Unicycler v.0.5.0 with default settings (3). All assemblies resulted in single sequences, which were identified as circular by Unicycler. No rotation of the genomes was performed. The genomes were annotated using the National Center for Biotechnology Information Prokaryotic Genome Annotation Pipeline (4 – 6) (Table 1).

**TABLE 1:** Genomic features of the sequenced strains

	Data for strain							
Feature^*a*^	BABAE-1	BABAE-2	BABAE-3	BABAE-4	BABAE-5	BABAE-6	BABAE-7	BABAE-8
GenBank accession no.	CP122561.1	CP122562.1	CP122563.1	CP122564.1	CP122565.1	CP122566.1	CP125880.1	CP125879.1
Short read accession no.	SRR24000767	SRR24000763	SRR24000761	SRR24000759	SRR24000757	SRR24000765	SRR24503426	SRR24503424
Long read accession no.	SRR24000766	SRR24000762	SRR24000760	SRR24000758	SRR24000756	SRR24000764	SRR24503425	SRR24503423
No. of short reads	1,743,764	1,656,040	1,636,978	1,584,960	1,887,080	2,129,740	1,582,282	1,813,072
No. of long reads	22,218	6,842	4,390	4,345	69,511	8,950	6,095	13,787
Long read *N*_50_ (bp)	8,417	18,526	18,879	16,607	8,992	16,297	21,005	10,906
Total sequence length (bp)	2,776,501	2,698,227	2,642,042	2,642,502	2,649,892	2,882,973	2,645,303	2,770,414
Genome coverage (x)	83.6	87.2	78.5	77.6	112.4	116.7	91.6	87.9
G + C content (%)	59.4	59.5	59.5	59.5	59.4	59.5	59.6	59.7
No. of CDSs	2,470	2,365	2,345	2,345	2,354	2,607	2,349	2,482
No. of rRNAs	6	6	6	6	6	6	6	6
No. of tRNAs	48	48	48	48	48	48	47	48
No. of ncRNAs	2	2	2	2	2	2	2	2

^*a*^ CDS, coding DNA sequence; ncRNA, noncoding RNAs.

**Fig 1:**
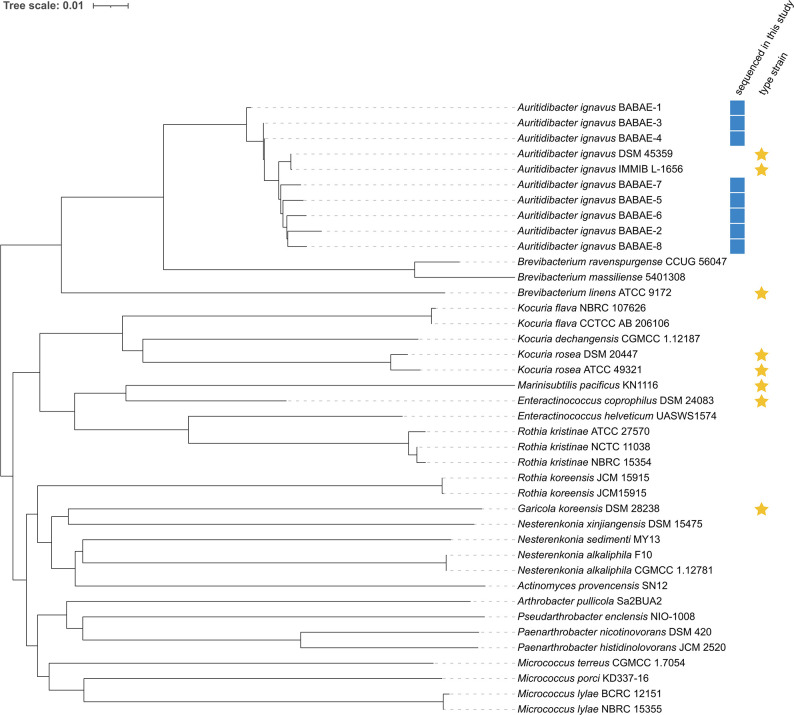
Phylogenetic tree inferred with FastME v.2.1.6.1 from Genome Blast Distance Phylogeny (GBDP) distances calculated from the assembled genomes and sequences and their closest relatives identified by TYGS. The branch lengths are scaled in terms of GBDP distance formula *d*_5_. The numbers above branches are GBDP pseudo-bootstrap support values of >60% from 100 replications, with an average branch support of 48.3%. The tree was rooted at the midpoint. Annotation of the tree was performed using iTOL v.6 (9).

Strain identification was confirmed by the Type (Strain) Genome Server (TYGS) (7, 8). The whole-genome sequence-based Genome Blast Distance Phylogeny tree calculated by TYGS revealed that all eight sequenced strains are closely related to the *A. ignavus* type strains DSM-45359 and IMMIB L-1656 (Fig. 1).

## Data Availability

The standardized strain descriptions and accession numbers are presented in Table 1; the genomic data are publicly available in DDBJ/ENA/GenBank under BioProject no. PRJNA949825 and in the Sequence Read Archive under accession no. SRP429893.
